# Influence of *NOD2* Variants on *Trichuris suis* ova Treatment Outcome in Crohn’s Disease

**DOI:** 10.3389/fphar.2018.00764

**Published:** 2018-07-16

**Authors:** Simon U. Jaeger, Elke Schaeffeler, Stefan Winter, Roman Tremmel, Jürgen Schölmerich, Nisar Malek, Eduard F. Stange, Matthias Schwab, Jan Wehkamp

**Affiliations:** ^1^Dr. Margarete Fischer Bosch Institute of Clinical Pharmacology, Stuttgart, Germany; ^2^University of Tübingen, Tübingen, Germany; ^3^Goethe University Frankfurt, Frankfurt, Germany; ^4^Department of Internal Medicine I, University of Tübingen, Tübingen, Germany; ^5^Department of Clinical Pharmacology, University of Tübingen, Tübingen, Germany; ^6^Department of Pharmacy and Biochemistry, University of Tübingen, Tübingen, Germany

**Keywords:** *Trichuris suis* ova, Crohn’s disease, NOD2, helminths, innate immunity

## Abstract

A recent randomized study of whipworm *Trichuris suis* ova (TSO) in ileal Crohn’s disease failed to demonstrate a clinical benefit compared to placebo after 12 weeks. Nonetheless, it has recently been shown that the spontaneous small intestinal inflammatory changes in *Nod2^-/-^* (Nucleotide-binding oligomerization domain 2) mice could be substantially ameliorated when these mice were colonized by *Trichuris muris.* Those and complementary epidemiologic findings in humans lead to the hypothesis that helminths may be advantageous only in patients carrying defective *NOD2* variants. Thus, 207 participants of the TSO trial were retrospectively genotyped for six functional *NOD2* genetic variants to evaluate whether the treatment outcome differed in patients carrying *NOD2* variants. We observed no significant association of the *NOD2* variants or their haplotypes with clinical outcome after TSO treatment.

## Introduction

Inflammatory bowel disease is most common in the Western world where helminthic colonization is rare. Along the lines of the so-called hygiene hypothesis, the presence of helminths including whipworms like *Trichuris trichiura* can be inversely correlated with the manifestation of immune-related conditions like Crohn’s disease. Yet, epidemiologic influences are difficult to entangle and possible mechanisms are still poorly understood.

The first indications for therapeutic efficacy of a helminth-based therapy came from two unblinded clinical trials which investigated viable *Trichuris suis* ova (TSO) both in Crohn’s disease and ulcerative colitis (Summers et al., 2005a,b). A study with a single administration of embryonated, viable ova of *Trichuris suis*, up to 7500 eggs, showed a good tolerability (Sandborn et al., 2013) but was not designed to evaluate response. This was addressed in the recent trial by Schölmerich et al. (2017), in which the clinical efficacy of a fortnightly suspension of three doses of TSO in mildly to moderately active, uncomplicated Crohn’s disease over 12 weeks were tested against placebo. In contrast to the previous studies, this first double-blind and randomized trial failed to demonstrate a clinical benefit for TSO compared to placebo, at least after 12 weeks of treatment.

The failure of the study might be explained by an insufficient therapy duration or by the unusually high placebo remission rate (42.9%). In addition, the authors considered that the prevention of disease relapse might have been a more suitable endpoint. Yet, it has to be acknowledged that the interplay between host immune function and *Trichuris* species is complex and remains insufficiently understood. Helminth products are known to modulate the host immune response, as for example, E/S glycans of the whipworm *T. suis* suppress LPS-induced cytokine production by intestinal epithelial cells and by dendritic cells. Furthermore, mucus production is increased (Hiemstra et al., 2014), which may facilitate helminth expulsion.

A role for the cytosolic pattern recognition receptor Nod2 (Nucleotide-binding oligomerization domain 2) in the host response to *Trichuris* species (in this case *Trichuris muris*) has been demonstrated in *colonic* epithelial cells in *Nod2^-/-^* mice. In the absence of Nod2, dendritic cell recruitment is impaired, which leads to an increased worm burden at day 21 alongside lower numbers of T cells in mesenteric lymph nodes (Bowcutt et al., 2014).

It has also been shown that Nod2*^-^*^/^*^-^* mice harbor several small intestinal abnormalities including increased proportion of IFN-γ expressing intraepithelial lymphocytes, goblet cell defects, and altered antimicrobial peptide expression. Nod2^-/-^ mice had a significantly different microbiome and the small intestinal changes depend on expansion of the Gram-negative anaerobic coccobacillus *Bacteroides vulgatus*. Accordingly, Nod2 prevents inflammation by restricting the pathological expansion of this important commensal bacterium (Ramanan et al., 2014).

In the recent study “Helminth infection promotes colonization resistance via type 2 immunity” by Ramanan et al. (2016), *Nod2^-/-^* mice were infected with ∼25 embryonated *T. muris* ova and evaluated at day 35 post infection. The small intestinal changes which arise in *Nod2*^-/-^ mice could be substantially ameliorated when these mice were colonized by *T. muris.* The helminth colonization induced a type 2 immunity response that favored the expansion of Clostridiales, which in turn inhibit colonization with the gram-negative anaerobic coccobacillus *B. vulgatus* and other Bacteroidales.

It was hypothesized from these findings and further epidemiologic evidence in humans that helminths may be beneficial only in patients with *NOD2* variants or proinflammatory Bacteroides species (Ramanan et al., 2016). The study design of the original TSO trial did not incorporate *NOD2* genetics, and thus it seemed worthwhile to investigate if outcome would in fact differ in those patients carrying genetic variants of *NOD2*.

## Materials and Methods

### Study Population

The study population is a subgroup from the abovementioned clinical trial (Schölmerich et al., 2017). Genetic analyses could be conducted in 207 patients who had signed an additional written informed consent at the beginning of the clinical trial, i.e., 60 patients treated with placebo, and 32, 62, and 53 patients treated with 250, 2500, and 7500 TSO, respectively. Sex, age, treatment group and values for CDAI at baseline and at week 12 visit were kindly made available by Dr. Ralph Mueller (Dr. Falk Pharma GmbH, Falk Foundation e.V., Freiburg, Germany).

### NOD2 Genotyping

Genomic DNA was isolated from whole blood using QIAamp DNA Blood Mini Kit (Qiagen, Hilden, Germany). Genotyping of genomic DNA for 2104C > T (rs2066844), 2722G > C (rs2066845), and 3016_3017insC (rs2066847) was performed using TaqMan technology (Life Technologies, Foster City, CA, United States), as described previously (Schwab et al., 2003). Genotype analyses of 802C > T (rs2066842), 1292C > T (rs104895431), and 2863G > A (rs5743291) was performed using predeveloped TagMan Assays (Life Technologies, Foster City, CA, United States) according to manufacturer’s protocol on a 7900 Real-Time PCR System. Results were analyzed by use of the Sequence Detection System (SDS) Software (Life Technologies, Foster City, CA, United States).

### Statistical Analysis

Statistical analyses were performed with R-3.4.1 and additional packages SNPassoc 1.9-2 and haplo.stats 1.7.7 (González et al., 2007; R Core Team, 2017; Sinnwell and Schaid, 2018). The primary endpoint remission (CDAI < 150 points at Week 12) and the secondary endpoint response (CDAI drop ≥ 100 at week 12 compared to baseline) were treated as binary variables (yes/no). Four genotyped patients in whom a CDAI value at week 12 was not available were excluded from the analysis. For each variant, multiple logistic regression analysis was applied to estimate the effect of the variant, the treatment as well as the variant–treatment interaction on the primary or secondary endpoint. Here, rs2066842 [Minor allele frequency (MAF) = 37.9%] was considered in the additive genetic model and the other five variants (MAF < 10%) in the dominant model. Moreover, type II ANOVA was applied to compute corresponding *P*-values (likelihood ratio test).

Haplotype estimation was based on an expectation–maximization (EM) algorithm (R package haplo.stats). The most frequent haplotype was defined as the “base” haplotype and haplotypes with a frequency below 5% were summarized in the “rare” haplotype. A generalized linear model of the haplo.stats package incorporating treatment as covariate without interaction term was used. Haplotypes were coded in the additive model. All tests were two-sided, *P*-value ≤ 0.05. Given *P*-values were not adjusted for multiple testing.

## Results and Discussion

Carriers of *NOD2* variants are already known to have an increased susceptibility to ileal Crohn’s disease (Hugot et al., 2001; Cleynen et al., 2016), and *NOD2* variants have been associated with an altered microbial composition (Kennedy et al., 2018) and with decreased expression of antimicrobial peptides (α-defensins), leading to a compromised antibacterial barrier (Wehkamp et al., 2004). In the recent publication by Ramanan et al. (2016) it was speculated that in the subset of patients carrying *NOD2* variants, helminth therapy could be more efficacious than in wild-type individuals. Therefore, we analyzed the impact of six important *NOD2* variants as a potential important confounder in the TSO trial, which evaluated outcome after 12 weeks of therapy with *T. suis* ova in Crohn’s disease.

**FIGURE 1:**
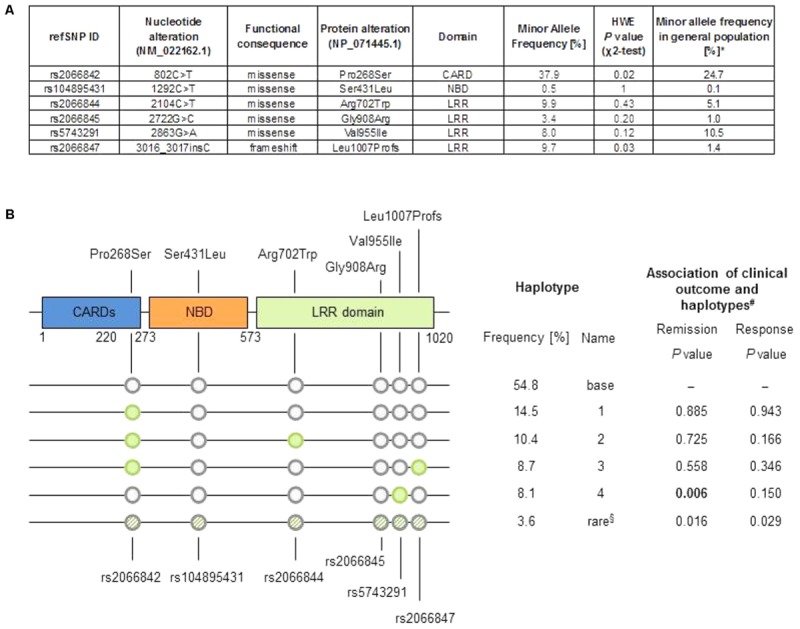
**(A)** Minor allele frequency in the cohort and general population, and *P*-value from χ^2^-test of Hardy–Weinberg equilibrium (HWE). ^∗^Data from 1000 Genomes Project (Europeans, Phase 3, build 144, http://www.internationalgenome.org/data). **(B)** Top: Schematic representation of the NOD2 protein, location of changes in amino acid sequence. LRR, leucine rich repeat; CARD, caspase recruitment domain; NBD, nuclear binding domain. Bottom: Graphic representation of haplotypes, sorted by frequency, as well as tabular summary of haplotype analysis result. §“Rare” haplotype summarizes all uncommon haplotypes (frequency < 5%). ^#^Based on logistic regression analysis comparing each haplotype versus the effect of the “base” haplotype (the most common haplotype).

We considered the most investigated functional variants Arg702Trp (rs2066844), Gly908Arg (rs2066845) and the frameshift variant Leu1007Profs (rs2066847) that leads to a truncated version of NOD2. In addition, three further coding variants were included in the analysis: the rare variant Ser431Leu (rs104895431) that does not activate NFκB in response to muramyl-dipeptide (Rivas et al., 2011), and the common variants Pro268Ser (rs2066842), with a MAF of 24.7%, and Val955Ile (rs5743291) with a MAF of 10.5% in Europeans (see **Figure 1A**).

The minor allele frequencies of the variants rs2066844, rs2066845, and rs2066847 in our Crohn’s disease cohort where higher compared to the general population, similarly as described in the literature (Hugot et al., 2001). The common variant rs2066842 and the frameshift variant rs2066847 slightly deviate from Hardy–Weinberg equilibrium, which is explained as *NOD2* is a known susceptibility locus for Crohn’s disease.

The influence of the treatment or the genetic variants on the primary endpoint of remission [defined as Crohn’s disease activity index (CDAI) < 150 points after 12 weeks] or the secondary endpoint of response (defined as ≥100 points drop in CDAI after 12 weeks compared to baseline) were evaluated with a multiple logistic regression analysis. No consistent significant association of both outcome measures with the six NOD2 variants or the treatment was observed: remission was significantly associated with the presence of rs5743291, but this could not be ascertained for response, which is clinically implausible. The effect of this variant was independent of the treatment group. No significant interaction effect between the variants and any treatment group was evident (**Table 1**).

**Table 1:** Prevalence of variants in treatment subgroups and logistic regression analyses for two clinical endpoints.

Genetic variant	Genotype	Treatment	*n* Remission or response/total (%)	*P*-values^∗^			
				Remission		Response	
				Main effect	Variant–treatment interaction effect	Main effect	Variant–treatment interaction effect
rs2066842 (Pro268Ser)	C/C	250	7/16 (44)	0.77	0.33	0.62	0.57
		2500	11/27 (41)				
		7500	15/23 (65)				
		placebo	8/19 (42)				
	C/T	250	2/10 (20)				
		2500	7/22 (32)				
		7500	8/21 (38)				
		placebo	15/26 (58)				
	T/T	250	4/5 (80)				
		2500	5/12 (42)				
		7500	2/5 (40)				
		placebo	5/11 (45)				
rs104895431 (Ser431Leu)	C/C	250	13/31 (42)	0.073	1	0.082	1
		2500	22/60 (37)				
		7500	24/48 (50)				
		placebo	28/56 (50)				
	C/T	250	–^#^				
		2500	1/1 (100)				
		7500	1/1 (100)				
		placebo	–^#^				
rs2066844 (Arg702Trp)	C/C	250	11/25 (44)	0.43	0.12	0.46	0.71
		2500	19/48 (40)				
		7500	23/40 (58)				
		placebo	21/46 (46)				
	C/T	250	2/6 (33)				
		2500	2/11 (18)				
		7500	2/9 (22)				
		placebo	6/9 (67)				
	T/T	250	–^#^				
		2500	2/2 (100)				
		7500	–^#^				
		placebo	1/1 (100)				
rs2066845 (Gly908Arg)	G/G	250	11/29 (38)	0.057	0.14	0.077	0.17
		2500	20/57 (35)				
		7500	24/46 (52)				
		placebo	26/54 (48)				
	G/C	250	2/2 (100)				
		2500	3/4 (75)				
		7500	1/3 (33)				
		placebo	1/1 (100)				
	C/C	250	–^#^				
		2500	–^#^				
		7500	–^#^				
		placebo	1/1 (100)				
rs5743291 (Val955Ile)	G/G	250	8/24 (33)	0.0051	0.92	0.35	0.55
		2500	18/52 (35)				
		7500	18/40 (45)				
		placebo	25/52 (48)				
	A/G	250	5/7 (71)				
		2500	3/6 (50)				
		7500	7/9 (78)				
		placebo	3/4 (75)				
	A/A	250	–^#^				
		2500	2/3 (67)				
		7500	–^#^				
		placebo	–^#^				
rs2066847 (Leu1007Profs)	–/–	250	11/28 (39)	0.69	0.23	0.3	0.49
		2500	19/50 (38)				
		7500	22/45 (49)				
		placebo	24/43 (56)				
	-/insC	250	2/3 (67)				
		2500	4/11 (36)				
		7500	2/3 (67)				
		placebo	4/10 (40)				
	insC/insC	250	–^#^				
		2500	–^#^				
		7500	1/1 (100)				
		placebo	0/3 (0)				

*^∗^Unadjusted *P*-value based on multiple logistic regression and type II ANOVA. ^#^No patients with corresponding variant-treatment combination in cohort.*

We further computed haplotypes in the cohort from the unphased genotype data. A pre-dominating haplotype (“base” haplotype) was found in 54.8% of cases, and additional four haplotypes showed a frequency of 8% or more (**Figure 1B**). The haplotype 4 differs from the “base” haplotype solely in rs5743291 and shows the same inconsistent association to the binary clinical endpoints as rs5743291 in the single variant analysis described above. Considering the study endpoints as continuous variables (i.e., CDAI count at follow-up or difference between CDAI count at follow-up and CDAI count at baseline) did not reveal any additional significant associations after correction for multiple testing.

Interestingly, the two patients who carried the rare functional variant rs104895431 had been randomized to the verum group and showed a remarkable benefit from treatment, i.e., a decrease in CDAI from 245 to 127 (patient received 2500 TSO), and from 346 to 60 points (7500 TSO). The latter patient simultaneously carried two further variants, rs2066842 and the frameshift variant rs2066847. Although this might be due to pure coincidence, the response to TSO treatment in the individual carrying three functional variants is noteworthy.

Yet in general, *NOD2* variants seem not to have a clinically meaningful impact on the outcome of the TSO trial, which can be explained by various reasons.

Firstly, as Nelson et al. (2016) have outlined, only rarely, even truly efficacious drugs will have a clinically meaningful genetic predictor of efficacy. In general, surrogate endpoints, e.g., a decrease in serum CRP, could help detecting differences in subgroups, but there was no overall response in terms of laboratory markers of intestinal inflammation in the original trial. Only blood eosinophilic counts, eosinophil derived neurotoxin in stools and the *T. suis* E/S antigen specific IgG showed dose-dependent effects (Schölmerich et al., 2017).

Secondly, the randomization process of the clinical study did not stratify according to genotype. That means, genetic variants and treatments are haphazardly distributed, e.g., heterozygous Leu1007Profs variants were present in 17% of placebo-treated individuals, but only in 8% of patients treated with 7500 *T. suis* ova.

Further, it has to be taken into account that the mostly heterozygous carriers of variants in the *NOD2* gene encountered in the TSO trial cannot be easily compared to a complete *NOD2* knock-out. Even in the experiments done with *Nod2*^-/-^ mice by Bowcutt et al. (2014) which demonstrated the importance of Nod2 in dentritic cell recruitment and early phases of the immune response, the knock-outs were ultimately able to expel *T. muris*. It would be interesting to know if specific variant models yielded similar effects in the animal model. The Arg702Trp, Gly908Arg, Val955Ile, and the frameshift variants affect the leucine-rich repeat domain of the NOD2 protein and hamper bacterial recognition, but may not abbrogate all functions of NOD2 relevant to intestinal inflammation.

The impact of NOD2 on microbial composition itself is still controversially discussed. For example, if *Nod2*^-/-^ and wild-type mice were co-housed, a *Nod2* genotype-specific effect on gut microbiota could no longer be demonstrated (Shanahan et al., 2014) – although coprophagy may have been a confounding variable. Other investigations have even shown that diet might be more relevant than genetic host factors (Carmody et al., 2015). To the contrast, Al Nabhani et al. (2016) did report *Nod2*-related differences in mucosal microbiota employing an embryo transfer strategy, which helped minimizing environmental and maternal influences.

Obviously, findings from *in vivo* mouse experiments and human pathophysiology might not always be in line, and *T. suis* adds even more complexity to this already intricate interaction. To conclude, although analyzing the genetic make-up of the well-characterized study population in this randomized trial was tempting, clinical efficacy of *T. suis* treatment was not better in carriers of six relevant *NOD2* variants.

## Ethics Statement

This study was carried out in accordance with the International Conference on Harmonisation (ICH) Guideline for Good Clinical Practice and was approved by independent ethic committees for each study center. All subjects gave written informed consent for original clinical trial and all patients included in this additional genetic analysis have signed a separate, optional informed consent.

## Author Contributions

SJ drafted the manuscript, organized and acquired data, took part in statistical analysis, interpreted data and was clinical investigator in original TSU trial. ES coordinated the genetic analysis, interpreted the data and critically revised the manuscript. SW and RT are biostatisticians who performed the statistical analysis and critically revised the manuscript. JS was the lead investigator of the original clinical trial and critically revised the manuscript. NM provided reagents and provided comments on drafts. EFS was investigator in the original clinical trial and critically revised the manuscript. MS contributed to conception and design of the study, provided funding and critically revised the manuscript. JW was principal investigator in the clinical trial, contributed to conception and design of the study, provided funding and critically revised the manuscript.

## Conflict of Interest Statement

The authors declare that the research was conducted in the absence of any commercial or financial relationships that could be construed as a potential conflict of interest.
